# Escore Ultrassonográfico de Congestão Venosa (VExUS) em Pacientes com Infarto do Miocárdio com Supradesnivelamento do Segmento ST para Predição da Mortalidade Intra-Hospitalar

**DOI:** 10.36660/abc.20250093

**Published:** 2025-07-01

**Authors:** Guilherme Pinheiro Machado, Guilherme Heiden Telo, João Pedro da Rosa Barbato, Marina Petersen Saadi, Gustavo Neves de Araujo, Angelo Chies, Julia Carvalho da Silva, Rodrigo Wainstein, Marco Wainstein

**Affiliations:** 1 Hospital de Clínicas de Porto Alegre Departamento de Cardiologia Porto Alegre RS Brasil Hospital de Clínicas de Porto Alegre, Departamento de Cardiologia, Porto Alegre, RS – Brasil; 2 Hospital da Unimed Grande Florianópolis Departamento de Cardiologia São José SC Brasil Hospital da Unimed Grande Florianópolis, Departamento de Cardiologia, São José, SC – Brasil; 3 Universidade Federal do Rio Grande do Sul Porto Alegre RS Brasil Universidade Federal do Rio Grande do Sul, Porto Alegre, RS – Brasil

**Keywords:** Síndrome coronariana aguda, Injúria Renal Aguda, Infarto do miocárdio

## Pontos-chave

1. Escore VExUS ≥1 está associado a maior mortalidade intra-hospitalar, ressaltando a importância da avaliação da congestão hemodinâmica em indivíduos com IAMCSST.
2. Escores VExUS mais altos se correlacionam com menor índice cardíaco, reforçando a relação entre congestão venosa e disfunção cardíaca no IAMCSST.
3. O VExUS pode ser uma ferramenta útil para identificar indivíduos de alto risco com IAMCSST, apoiando a necessidade de uma avaliação hemodinâmica abrangente.

## Introdução

Nos últimos anos, o uso da ultrassonografia à beira do leito (*point-of-care ultrasound*, POCUS) cresceu exponencialmente em diversos contextos clínicos, incluindo unidades de terapia intensiva e departamentos de emergência. Essa modalidade tem se mostrado particularmente valiosa em pacientes gravemente enfermos, por oferecer uma avaliação mais sensível e não invasiva do estado hemodinâmico e volêmico. Mais recentemente, o escore ultrassonográfico de congestão venosa (*Venous Excess Ultrasound*, VExUS) ganhou destaque por sua capacidade de prever lesão renal aguda (LRA) tanto em pacientes no pós-operatório de cirurgia cardíaca quanto em pacientes críticos, auxiliando no manejo da terapia diurética. Uma versão simplificada do método VExUS — com foco nos padrões de fluxo Doppler das veias hepática e porta — demonstrou ser eficaz na detecção da congestão venosa. Isso facilita as decisões sobre manejo de fluidos e melhora a viabilidade e a reprodutibilidade da avaliação à beira do leito.^1^

Como a LRA afeta um número substancial de pacientes com síndrome coronariana aguda (SCA) e está fortemente associada à mortalidade tanto a curto quanto a longo prazo, a possível utilidade do escore VExUS nesse contexto merece investigação adicional. Fatores de risco já estabelecidos para LRA nessa população incluem idade avançada, doença renal crônica (DRC) pré-existente, infarto extenso da parede anterior e alta classe de Killip.^2,3^ Embora o escore VExUS tenha se mostrado eficaz na avaliação da congestão e na identificação da síndrome cardiorrenal, seu papel no cenário da SCA ainda não está claramente definido.

O único estudo publicado até o momento que avaliou o VExUS em pacientes com SCA sugeriu que um escore VExUS ≥1 poderia predizer a ocorrência de LRA, mas não demonstrou associação com a mortalidade — provavelmente devido ao pequeno tamanho da amostra.^4^ Assim, o presente estudo tem como objetivo investigar a relação entre os escores VExUS e a mortalidade intra-hospitalar em uma coorte maior de pacientes diagnosticados exclusivamente com infarto do miocárdio (IAM) com supradesnivelamento do segmento ST (IAMCSST).

## Métodos

### Desenho do estudo e população

Este foi um registro de coorte prospectiva com pacientes com IAMCSST admitidos para intervenção coronária percutânea primária (ICP) entre julho de 2022 e julho de 2023. Foram incluídos consecutivamente pacientes adultos (≥18 anos) com suspeita de IAMCSST, com base na presença de dor torácica típica em repouso associada a supradesnivelamento do segmento ST ou outras alterações eletrocardiográficas compatíveis com os critérios diagnósticos para IAMCSST. O diagnóstico e o tratamento do IAMCSST foram definidos de acordo com as diretrizes mais recentes disponíveis no momento da inclusão.^5^

### Critérios de exclusão

Foram excluídos os pacientes que se apresentaram mais de 12 horas após o início dos sintomas, que realizaram a avaliação por POCUS mais de 24 horas após a admissão ou que apresentavam janela acústica desfavorável.

### Desfechos do estudo

O desfecho primário foi a mortalidade intra-hospitalar. O desfecho secundário foi a ocorrência de LRA. O acompanhamento intra-hospitalar foi definido do momento da admissão até a alta hospitalar ou o óbito.

### Avaliação por ultrassonografia

A POCUS foi realizada nas primeiras 24 horas após a admissão, no departamento de emergência ou na unidade coronariana, utilizando um aparelho de ultrassonografia portátil (SonoSite, Bothell, Washington, EUA) equipado com transdutor setorial de 2,5 MHz. Os sinais vitais foram registrados no momento da avaliação. Dois investigadores treinados realizaram todos os exames de ultrassonografia, que duraram no máximo 3 minutos e não atrasaram o tempo porta-balão quando realizados antes da ICP.

Os pacientes foram avaliados em posição supina. A avaliação incluiu a medida do diâmetro da veia cava inferior e a análise do fluxo Doppler das veias hepática e porta, com traçado eletrocardiográfico simultâneo. A classificação do VExUS variou de 0 (sem congestão) a 3 (congestão grave).

### Definição de LRA

A LRA foi definida como um aumento da creatinina sérica ≥0,3 mg/dl (≥26,5 µmol/l) em até 48 horas, ou um aumento ≥1,5 vezes em relação ao valor basal (conhecido ou presumido como ocorrido nos 7 dias anteriores), ou ainda diurese <0,5 ml/kg/h por 6 horas.^6^ Métodos e definições adicionais foram descritos previamente.^7^

### Cálculo do tamanho amostral

Assumindo uma incidência de 10% de mortalidade intra-hospitalar, uma amostra de 132 pacientes proporcionaria um poder estatístico de 80% para detectar uma área sob a curva ROC (AUC) de 0,75, com um alfa bilateral de 0,05.

### Análise estatística

As variáveis contínuas foram apresentadas como média ± desvio padrão ou mediana e intervalo interquartil (IIQ), conforme apropriado. As variáveis categóricas foram apresentadas como contagens absolutas e percentuais. As comparações entre os grupos foram realizadas utilizando o teste t de Student para amostras independentes nas variáveis contínuas e o teste do qui-quadrado (χ^2^) para variáveis categóricas.

As curvas ROC foram utilizadas para avaliar o poder discriminatório do escore VExUS e da classificação de Killip, com os resultados expressos como estatísticas c. As AUCs foram comparadas utilizando o teste de DeLong.

Para identificar preditores de mortalidade intra-hospitalar, foi utilizado um modelo linear generalizado com regressão logística binária e função de ligação logit. O modelo multivariado incluiu variáveis clínicas associadas ao desfecho primário, como idade, sexo, IAM de parede anterior, DRC, VExUS ≥1 e doença multivascular (≥três vasos).

As comparações entre curvas ROC foram realizadas utilizando o MedCalc Statistical Software, versão 14.8.1 (MedCalc Software bvba, Ostend, Bélgica). Todas as demais análises estatísticas foram realizadas com o IBM SPSS Statistics para Windows, versão 29.0 Armonk, NY: IBM Corp.

## Resultados

**Figura 1:**
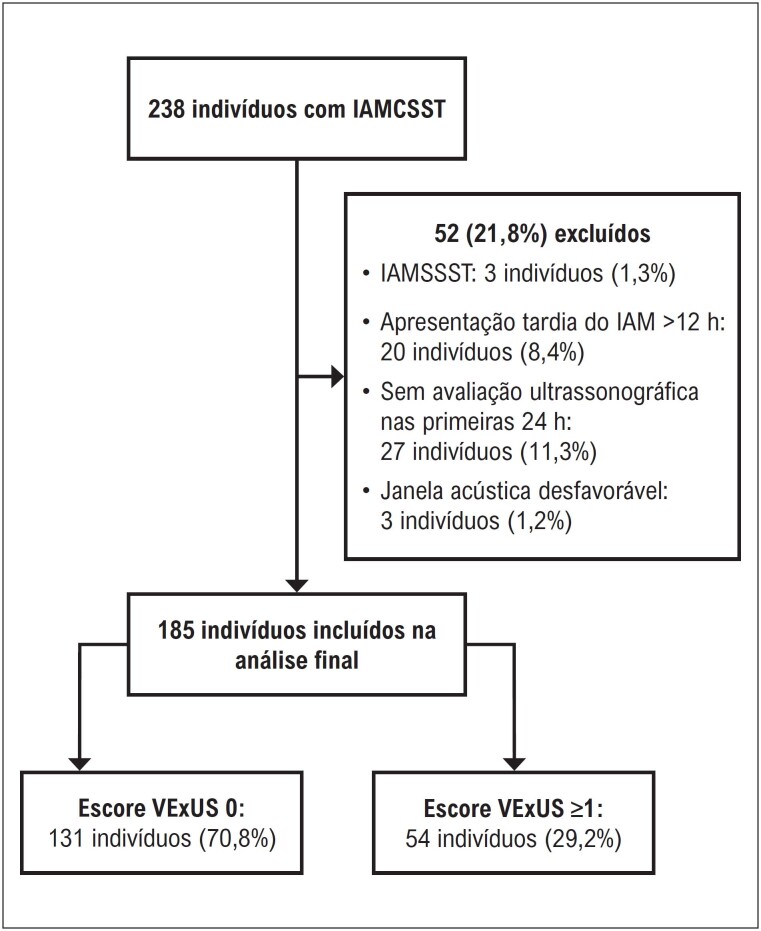
Amostra do estudo e alocação dos grupos.

Entre julho de 2022 e julho de 2023, um total de 238 pacientes foi triado, e 185 foram incluídos na análise final. Cinquenta e três pacientes (22,3%) foram excluídos devido a IAM sem supradesnivelamento do segmento ST (IAMSSST) (três pacientes [1,3%]), apresentação tardia do IAM (mais de 12 horas após o início dos sintomas) (20 pacientes [8,4%]), ausência de avaliação ultrassonográfica nas primeiras 24 horas após a admissão (27 pacientes [11,3%]) ou janela acústica desfavorável (três pacientes [1,2%]). A Figura 1 apresenta o fluxograma do estudo.

A média de idade da população do estudo foi de 62±11 anos, e 70% eram do sexo masculino. Hipertensão arterial esteve presente em 56% dos pacientes, diabetes em 28% e DRC em 7%. Na admissão, 43% dos pacientes apresentavam IAM de parede anterior, e 13% foram classificados como Killip classe 3 ou 4.

A taxa global de mortalidade intra-hospitalar foi de 7,6%, e a incidência de LRA foi de 8,6%. As características clínicas basais dos pacientes estão resumidas na Tabela 1.

**Tabela 1:** Características basais

Variável		Total (n=185)	Escore VExUS 0 (n=131)	Escore VExUS ≥1 (n=54)	Valor de p
Idade, anos		61,9 (11,3)	62,0 (10,7)	61,8 (12,8)	0,95
Sexo masculino, n (%)		130 (70,3)	94 (71,8)	36 (66,7)	0,48
IMC, kg/m²		27,0 (5,0)	27,1 (5,0)	26,7 (5,0)	0,63
Hipertensão, n (%)		104 (56,2)	76 (58,0)	28 (51,9)	0,44
Diabetes, n (%)		52 (28,1)	37 (28,2)	15 (27,2)	0,88
DRC, n (%)		13 (7,0)	9 (6,9)	4 (7,4)	0,89
Tabagismo, atual ou anterior, n (%)		102 (55,1)	86 (51,9)	34 (62,9)	0,19
História familiar de DAC, n (%)		16 (8,9)	10 (7,9)	6 (11,1)	0,49
ICC prévia, n (%)		5 (2,7)	4 (3,1)	1 (1,9)	0,64
IAM prévio, n (%)		24 (13,0)	15 (11,5)	9 (16,7)	0,33
Parada cardiorrespiratória, n (%)		13 (7,0)	4 (3,1)	7 (13,0)	0,04
IAM de parede anterior, n (%)		80 (43,2)	62 (47,3)	18 (33,3)	0,08
**Classe de Killip, n (%)**					<0,001
	Classe I	131 (70,8)	104 (79,4)	27 (50,0)	
	Classe II	30 (16,2)	19 (14,5)	11 (20,4)	
	Classe III	6 (3,2)	2 (1,5)	4 (7,4)	
	Classe IV	18 (9,7)	6 (4,6)	12 (22,2)	
Tempo entre sintomas e chegada, min		300 (187-420)	300 (195-420)	300 (183-411)	0,81
Tempo porta-balão, min		53 (34-76)	54 (31-75)	49 (40-87)	0,59
Escore TIMI		4 (2-4)	3 (1-5)	5 (3-6)	<0,001
Tempo de internação, dias		6 (5-9)	6 (5-8)	7,5 (5-12)	0,02
**Características do procedimento**					
**Artéria culpada, n (%)**					0,36
	Coronária direita (CD)	77 (42,3)	60 (46,2)	17 (32,7)	
	Descendente anterior (DA)	19 (10,4)	14 (10,7)	5 (9,6)	
	Circunflexa (Cx)	13 (7,2)	11 (6,9)	2 (3,8)	
	Outra	73 (40,1)	45 (34,6)	28 (53,8)	
Doença multivascular, n (%)		51 (27,6)	32 (24,4)	19 (35,2)	0,13
Acesso femoral, n (%)		38 (21,0)	24 (18,8)	14 (26,4)	0,24
Sucesso angiográfico, n (%)		172 (95,6)	122 (95,3)	50 (96,2)	0,80

*CD: artéria coronária direita; Cx: artéria circunflexa; DA: artéria descendente anterior; DAC: doença arterial coronariana; DRC: doença renal crônica; IAM: infarto agudo do miocárdio; ICC: insuficiência cardíaca; IMC: índice de massa corporal; TIMI:* thrombolysis in myocardial infarction.

**Figura 2:**
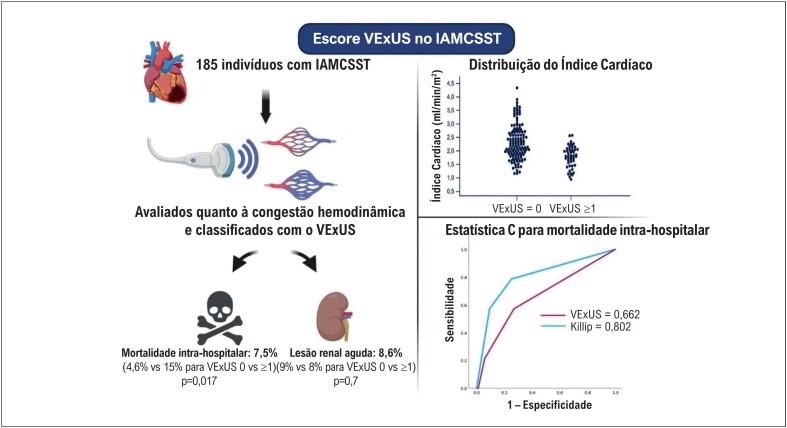
Sistema de pontuação VExUS e sua associação com mortalidade hospitalar e lesão renal aguda (à esquerda). Distribuição do índice cardíaco por grupo VExUS (acima, à direita) e curvas ROC para mortalidade hospitalar comparando VExUS e a classificação de Killip (abaixo, à direita). Figura criada com BioRender.com.

Os principais achados da análise estão ilustrados na Figura 2. A distribuição dos indivíduos conforme os graus do escore VExUS foi a seguinte: VExUS 0 em 131 indivíduos (70,8%), VExUS 1 em 41 (22,2%), VExUS 2 em 11 (5,9%) e VExUS 3 em dois (1,1%). Observou-se um aumento progressivo da mortalidade com escores VExUS mais altos: 4,6% para VExUS 0 (seis óbitos), 12,2% para VExUS 1 (cinco óbitos) e 27,3% para VExUS 2 (três óbitos).

A AUC para mortalidade intra-hospitalar foi de 0,662 (IC 95%, 0,502-0,823) para o escore VExUS e de 0,802 (IC 95%, 0,667-0,938) para a classificação de Killip (p=0,16 para comparação entre AUCs). Quando a classificação de Killip e o escore VExUS foram combinados para identificar indivíduos de alto risco, a AUC aumentou para 0,855. Nesse modelo combinado, as classes III e IV de Killip permaneceram inalteradas, enquanto foi criada uma nova categoria intermediária, composta por indivíduos com Killip classe I e VExUS ≥1, e aqueles com Killip classe II e VExUS 0.

Não houve diferença significativa na incidência de LRA entre os grupos VExUS. No entanto, indivíduos com VExUS ≥1 apresentaram maior frequência de IAM de ventrículo direito (16,7% vs 6,9%; p=0,04), menor índice cardíaco (1,7±0,3 ml/min/m^2^ vs 2,3±0,6 ml/min/m^2^; p<0,001) e menor fração de ejeção do ventrículo esquerdo (45%±12 vs 49%±10; p=0,008).

No modelo de regressão logística multivariada, idade (OR=1,07; IC 95%, 1,01-1,14; p=0,01), IAM de parede anterior (OR=5,0; IC 95%, 1,34-18,98; p=0,01) e VExUS ≥1 (OR=5,2; IC 95%, 1,48-18,76; p=0,01) estiveram associados de forma independente à mortalidade intra-hospitalar.

## Discussão

Neste estudo de coorte prospectiva que avaliou o escore VExUS em indivíduos com IAMCSST submetidos à ICP primária, a presença de VExUS ≥1 esteve associada a maior mortalidade intra-hospitalar e menor índice cardíaco. Até onde sabemos, este é o maior estudo realizado até o momento que avalia o VExUS no contexto da SCA, e o primeiro com foco exclusivo em IAMCSST. É também o primeiro a demonstrar uma associação entre o escore VExUS e a mortalidade nessa população específica.

O escore VExUS tem se consolidado como uma ferramenta valiosa para a avaliação da congestão venosa, com possíveis implicações na estratificação do risco de mortalidade no IAMCSST. Sua capacidade de quantificar a sobrecarga hemodinâmica por meio de um exame não invasivo oferece um método promissor para identificar indivíduos com maior risco de desfechos adversos. No entanto, embora o VExUS pareça útil na predição de mortalidade ao captar diferentes graus de congestão sistêmica, seu papel na avaliação da LRA no IAMCSST pode ser limitado. Essa limitação provavelmente decorre da fisiopatologia multifatorial da LRA nesse contexto, que envolve não apenas a congestão venosa, mas também lesão renal isquêmica, nefropatia induzida por contraste e diversos fatores inflamatórios e hemodinâmicos sistêmicos.^2,3^ Portanto, confiar exclusivamente no VExUS para predizer LRA pode levar à negligência de importantes contribuintes para a disfunção renal no IAMCSST, reduzindo sua utilidade para esse desfecho específico. Nossos achados diferem dos de Viana-Rojas et al.,^4^ provavelmente devido às diferenças nas populações estudadas. Enquanto a análise deles incluiu indivíduos com angina instável e IAMSSST, nosso estudo concentrou-se exclusivamente em IAMCSST. Ainda assim, ambos os estudos apoiam o uso de escores VExUS alterados como marcadores de risco significativos para desfechos adversos na SCA. Embora outras ferramentas baseadas em ultrassonografia — como o ultrassom pulmonar e o tempo de integral de velocidade na via de saída do ventrículo esquerdo^7,8^ — possam apresentar desempenho superior na identificação de indivíduos de alto risco quando comparadas à avaliação clínica isolada, nossos achados sugerem que a integração do VExUS à classificação de Killip melhora ainda mais a acurácia preditiva, conforme demonstrado pelo aumento da AUC. Isso indica que o VExUS pode atuar como um complemento valioso na estratificação precoce do risco, especialmente no contexto de IAM de ventrículo direito. No entanto, essa hipótese exige investigação e validação adicionais em coortes maiores, particularmente naquelas com maior prevalência de acometimento do ventrículo direito.

Este estudo apresenta várias limitações. Primeiramente, seu delineamento unicêntrico e o tamanho amostral relativamente pequeno podem ter limitado o poder estatístico e a precisão do modelo de regressão. No entanto, é importante destacar que se tratou de um registro de indivíduos consecutivos e não selecionados com IAMCSST admitidos em um centro de referência terciário, o que favorece a generalização dos resultados para contextos clínicos semelhantes. Até o momento, este é o primeiro e maior estudo a avaliar o escore VExUS especificamente em indivíduos com IAMCSST e o primeiro a demonstrar uma associação entre o VExUS e a mortalidade intra-hospitalar nessa população. Ainda assim, estudos multicêntricos futuros são necessários para confirmar e expandir esses achados.

## Conclusão

O VExUS demonstra ser uma ferramenta promissora para identificar indivíduos de alto risco e prever a mortalidade intra-hospitalar após IAMCSST. Esses achados ressaltam o valor de uma avaliação hemodinâmica abrangente na estratificação prognóstica precoce dessa população.

## Data Availability

Todo o conjunto de dados que dá suporte aos resultados deste estudo está disponível mediante solicitação ao autor correspondente, Guilherme Pinheiro Machado, mediante solicitação razoável. O conjunto de dados não está publicamente disponível por conterem informações quem podem comprometer a privacidade dos participantes da pesquisa.

## References

[1] Bhardwaj V, Vikneswaran G, Rola P, Raju S, Bhat RS, Jayakumar A (2020). Combination of Inferior Vena Cava Diameter, Hepatic Venous Flow, and Portal Vein Pulsatility Index: Venous Excess Ultrasound Score (VEXUS Score) in Predicting Acute Kidney Injury in Patients with Cardiorenal Syndrome: A Prospective Cohort Study. Indian J Crit Care Med.

[2] Wang C, Pei YY, Ma YH, Ma XL, Liu ZW, Zhu JH (2019). Risk Factors for Acute Kidney Injury in Patients with Acute Myocardial Infarction. Chin Med J.

[3] Shacham Y, Leshem-Rubinow E, Steinvil A, Assa EB, Keren G, Roth A (2014). Renal Impairment According to Acute Kidney Injury Network Criteria among ST Elevation Myocardial Infarction Patients Undergoing Primary Percutaneous Intervention: A Retrospective Observational Study. Clin Res Cardiol.

[4] Viana-Rojas JA, Argaiz E, Robles-Ledesma M, Arias-Mendoza A, Nájera-Rojas NA, Alonso-Bringas AP (2023). Venous Excess Ultrasound Score and Acute Kidney Injury in Patients with Acute Coronary Syndrome. Eur Heart J Acute Cardiovasc Care.

[5] Byrne RA, Rossello X, Coughlan JJ, Barbato E, Berry C, Chieffo A (2023). 2023 ESC Guidelines for the Management of Acute Coronary Syndromes. Eur Heart J.

[6] Khwaja A (2012). KDIGO Clinical Practice Guidelines for Acute Kidney Injury. Nephron Clin Pract.

[7] Machado GP, Telo GH, Araújo GN, Barbato JPR, Amon A, Martins A (2024). A Combination of Left Ventricular Outflow Tract Velocity Time Integral and Lung Ultrasound to Predict Mortality in ST Elevation Myocardial Infarction. Intern Emerg Med.

[8] Araújo GN, Silveira AD, Scolari FL, Custodio JL, Marques FP, Beltrame R (2020). Admission Bedside Lung Ultrasound Reclassifies Mortality Prediction in Patients with ST-Segment-Elevation Myocardial Infarction. Circ Cardiovasc Imaging.

