# Supplementary material for: Escore Ultrassonográfico de Congestão Venosa (VExUS) em Pacientes com Infarto do Miocárdio com Supradesnivelamento do Segmento ST para Predição da Mortalidade Intra-Hospitalar
Source: Arq Bras Cardiol. 2025 Jul 1;122(6):e20250093. [Article in Portuguese] doi: 10.36660/abc.20250093 (PMC12269907; doi:10.36660/abc.20250093)
Supplement: Translation

# Translation: Venous Excess Ultrasound (VExUS) Score in Patients with ST-Elevation Myocardial Infarction to Predict In-Hospital Mortality

**DOI:** 10.36660/abc.20250093i

**Authors:** Guilherme Pinheiro Machado, Guilherme Heiden Telo, João Pedro da Rosa Barbato, Marina Petersen Saadi, Gustavo Neves de Araujo, Angelo Chies, Julia Carvalho da Silva, Rodrigo Wainstein, Marco Wainstein

**Affiliations:** 1 Porto Alegre RS Brazil Hospital de Clínicas de Porto Alegre, Departamento de Cardiologia, Porto Alegre, RS – Brazil; 2 São José SC Brazil Hospital da Unimed Grande Florianópolis, Departamento de Cardiologia, São José, SC – Brazil; 3 Porto Alegre RS Brazil Universidade Federal do Rio Grande do Sul, Porto Alegre, RS – Brazil

**Keywords:** Acute Coronary Syndrome, Acute Kidney Injury, Myocardial Infarction

## Key points

1. VExUS scores ≥1 are associated with increased in-hospital mortality, highlighting the importance of assessing hemodynamic congestion in individuals with STEMI.
2. Higher VExUS scores correlate with significantly lower cardiac index, reinforcing the link between venous congestion and impaired cardiac function in STEMI.
3. VExUS may be a valuable tool for identifying high-risk individuals with STEMI, supporting the need for comprehensive hemodynamic evaluation.

## Introduction

In recent years, the use of point-of-care ultrasound (POCUS) has grown exponentially for bedside assessments in a variety of clinical settings, including intensive care units and emergency departments. This modality has proven particularly valuable in critically ill patients, offering a more sensitive and noninvasive evaluation of hemodynamic and volume status. More recently, the Venous Excess Ultrasound (VExUS) scoring system has gained attention for its ability to predict acute kidney injury (AKI) in both postcardiac surgery and critically ill patients, aiding in the management of diuretic therapy. A simplified version of the VExUS approach — focused on hepatic and portal vein Doppler flow patterns — has demonstrated reliable detection of venous congestion. This facilitates fluid management decisions while improving feasibility and reproducibility at the bedside.^1^

Since AKI affects a substantial number of patients with acute coronary syndrome (ACS) and is strongly associated with both short- and long-term mortality, the potential utility of the VExUS scoring system in this context merits further investigation. Established risk factors for AKI in this population include advanced age, preexisting chronic kidney disease (CKD), extensive anterior infarction, and a high Killip class.^2,3^ Although the VExUS score has proven effective in assessing congestion and identifying cardiorenal syndrome, its role in the ACS setting remains unclear.

The only study to date that evaluated VExUS in ACS patients suggested that a VExUS score of ≥1 might predict AKI, but it did not demonstrate an association with mortality — likely due to the study's small sample size.^4^ Accordingly, the present study aims to investigate the relationship between VExUS scores and in-hospital mortality in a larger cohort of patients diagnosed exclusively with ST-elevation myocardial infarction (MI) (STEMI).

## Methods

### Study design and population

This was a prospective cohort registry of patients with STEMI admitted for primary percutaneous coronary intervention (PCI) between July 2022 and July 2023. Consecutive adult patients (≥18 years old) with suspected STEMI were eligible for inclusion based on the presence of typical chest pain at rest associated with ST-segment elevation or other electrocardiography (ECG) abnormalities meeting diagnostic criteria for STEMI. STEMI diagnosis and treatment were defined according to the most recent guidelines available at the time of enrollment.^5^

### Exclusion criteria

Patients were excluded if they presented more than 12 hours after symptom onset, underwent POCUS assessment more than 24 hours after admission, or had an unfavorable acoustic window.

### Study endpoints

The primary endpoint was in-hospital mortality. The secondary endpoint was the occurrence of AKI. In-hospital follow-up was defined from the date of admission to the date of discharge or death.

### Ultrasound assessment

POCUS was performed within 24 hours of admission in the emergency department or coronary care unit using a portable ultrasound device (SonoSite, Bothell, Washington, USA) equipped with a 2.5-MHz sectorial transducer. Vital signs were recorded at the time of evaluation. Two trained investigators performed all ultrasound examinations, which lasted no longer than 3 min and did not delay door-to-balloon time when performed prior to PCI.

Patients were evaluated in the supine position. The assessment included measurement of the inferior vena cava diameter and Doppler flow analysis of the hepatic and portal veins, with simultaneous ECG tracing. VExUS grading ranged from 0 (no congestion) to 3 (severe congestion).

### Definition of AKI

AKI was defined as an increase in serum creatinine of ≥0.3 mg/dL (≥26.5 µmol/L) within 48 hours, or an increase of ≥1.5 times the baseline (known or presumed to have occurred within the prior 7 days), or urine output <0.5 mL/kg/h for 6 h.^6^ Additional methods and definitions have been described previously.^7^

### Sample size calculation

Assuming a 10% incidence of in-hospital mortality, a sample of 132 patients would provide 80% statistical power to detect an area under the receiver operating characteristic (ROC) curve (AUC) of 0.75, with a two-sided *α* of 0.05.

### Statistical analysis

Continuous variables are presented as mean ± standard deviation or median and interquartile range (IQR), as appropriate. Categorical variables are presented as counts and percentages. Comparisons between groups were made using independent samples Student's *t*-test for continuous variables and the chi-square (*χ*^2^) test for categorical variables.

ROC curves were used to evaluate the discriminatory power of the VExUS score and Killip classification, with results expressed as c-statistics. AUCs were compared using the DeLong test.

To identify predictors of in-hospital mortality, we performed a generalized linear model with binary logistic regression and a logit link function. The multivariable model included clinical variables associated with the primary outcome, such as age, sex, anterior wall MI, CKD, VExUS ≥1, and multivessel disease (≥three vessels).

ROC curve comparisons were conducted using MedCalc Statistical Software version 14.8.1 (MedCalc Software bvba, Ostend, Belgium). All other statistical analyses were performed using IBM SPSS Statistics for Windows, version 29.0 Armonk, NY: IBM Corp.

## Results

Between July 2022 and July 2023, a total of 238 patients were screened, and 185 were included in the final analysis. Fifty-three patients (22.3%) were excluded due to non-STEMI (NSTEMI) (three patients [1.3%]), late presentation of MI more than 12 h after symptom onset (20 patients [8.4%]), absence of ultrasound assessment within 24 h of admission (27 patients [11.3%]), or an unfavorable acoustic window (three patients [1.2%]). Figure 1 shows the study flow diagram.

**Figure 1:** Study sample and group allocation.

The mean age of the study population was 62±11 years, and 70% were male. Hypertension was present in 56% of patients, diabetes in 28%, and CKD in 7%. At admission, 43% of patients had anterior wall MI, and 13% were classified as Killip class 3 or 4.

The overall in-hospital mortality rate was 7.6%, and the incidence of AKI was 8.6%. Baseline patient characteristics are summarized in Table 1.

**Table 1:** Baseline characteristics

Variable		Overall (n=185)	VExUS grade 0 (n=131)	VExUS grade ≥1 (n=54)	p-value
Age, years		61.9 (11.3)	62.0 (10.7)	61.8 (12.8)	0.95
Male sex, n (%)		130 (70.3)	94 (71.8)	36 (66.7)	0.48
BMI, kg/m²		27.0 (5.0)	27.1 (5.0)	26.7 (5.0)	0.63
Hypertension, n (%)		104 (56.2)	76 (58.0)	28 (51.9)	0.44
Diabetes, n (%)		52 (28.1)	37 (28.2)	15 (27.2)	0.88
CKD, n (%)		13 (7.0)	9 (6.9)	4 (7.4)	0.89
Smoking, current or previous, n (%)		102 (55.1)	86 (51.9)	34 (62.9)	0.19
Family history of CAD, n (%)		16 (8.9)	10 (7.9)	6 (11.1)	0.49
Previous HF, n (%)		5 (2.7)	4 (3.1)	1 (1.9)	0.64
Previous MI, n (%)		24 (13.0)	15 (11.5)	9 (16.7)	0.33
Sudden cardiac arrest, n (%)		13 (7.0)	4 (3.1)	7 (13.0)	0.04
Anterior MI, n (%)		80 (43.2)	62 (47.3)	18 (33.3)	0.08
**Killip class, n (%)**					<0.001
	Class I	131 (70.8)	104 (79.4)	27 (50.0)	
	Class II	30 (16.2)	19 (14.5)	11 (20.4)	
	Class III	6 (3.2)	2 (1.5)	4 (7.4)	
	Class IV	18 (9.7)	6 (4.6)	12 (22.2)	
Symptoms-to-door time, min		300 (187-420)	300 (195-420)	300 (183-411)	0.81
Door-to-balloon time, min		53 (34-76)	54 (31-75)	49 (40-87)	0.59
TIMI score		4 (2-4)	3 (1-5)	5 (3-6)	<0.001
Length of stay, days		6 (5-9)	6 (5-8)	7.5 (5-12)	0.02
**Procedural characteristics**					
**Culprit vessel, n (%)**					0.36
	RCA	77 (42.3)	60 (46.2)	17 (32.7)	
	LAD	19 (10.4)	14 (10.7)	5 (9.6)	
	LCx	13 (7.2)	11 (6.9)	2 (3.8)	
	Other	73 (40.1)	45 (34.6)	28 (53.8)	
Multivessel disease, n (%)		51 (27.6)	32 (24.4)	19 (35.2)	0.13
Femoral access, n (%)		38 (21.0)	24 (18.8)	14 (26.4)	0.24
Angiographic success, n (%)		172 (95.6)	122 (95.3)	50 (96.2)	0.80

BMI: body mass index; CAD: coronary artery disease; CKD: chronic kidney disease; HF: heart failure; LAD: left anterior descending artery; LCx: left circumflex artery; MI: myocardial infarction; RCA: right coronary artery; TIMI: thrombolysis in myocardial infarction.

The main findings of our analysis are illustrated in Figure 2. The distribution of individuals across VExUS grades was as follows: VExUS 0 in 131 individuals (70.8%), VExUS 1 in 41 (22.2%), VExUS 2 in 11 (5.9%), and VExUS 3 in two (1.1%). A stepwise increase in mortality was observed with higher VExUS grades: 4.6% for VExUS 0 (six deaths), 12.2% for VExUS 1 (five deaths), and 27.3% for VExUS 2 (three deaths).

**Figure 2:** VExUS scoring system and its association with in-hospital mortality and acute kidney injury (left). Cardiac index distribution by VExUS group (top right) and ROC curves for in-hospital mortality comparing VExUS and Killip classification (bottom right). Figure created with BioRender.com.

The AUC for in-hospital mortality was 0.662 (95% CI, 0.502-0.823) for VExUS and 0.802 (95% CI, 0.667-0.938) for the Killip classification (p=0.16 for AUC comparison). When Killip classification and VExUS were combined to identify high-risk individuals, the AUC increased to 0.855. In this combined model, Killip classes III and IV remained unchanged, while a new intermediate category was created, comprising individuals with Killip class I and VExUS ≥1, and those with Killip class II and VExUS 0.

There was no significant difference in the incidence of AKI across VExUS groups. However, individuals with VExUS ≥1 had a higher frequency of right ventricular MI (16.7% vs 6.9%, p=0.04), lower cardiac index (1.7±0.3 mL/min/m^2^ vs 2.3±0.6 mL/min/m^2^; p<0.001), and lower left ventricular ejection fraction (45%±12 vs 49%±10; p=0.008).

In the multivariate logistic regression model, age (OR=1.07; 95% CI, 1.01-1.14; p=0.01), anterior wall MI (OR=5.0; 95% CI, 1.34-18.98; p=0.01), and VExUS ≥1 (OR=5.2; 95% CI, 1.48-18.76; p=0.01) were independently associated with in-hospital mortality.

## Discussion

In this prospective cohort study evaluating the VExUS scoring system in individuals with STEMI undergoing primary PCI, the presence of VExUS ≥1 was associated with higher in-hospital mortality and lower cardiac index. To our knowledge, this is the largest study to date assessing VExUS in ACS, and the first focused exclusively on STEMI. It is also the first to demonstrate an association between VExUS and mortality in this specific population.

The VExUS scoring system has emerged as a valuable tool for assessing venous congestion, with potential implications for mortality risk stratification in STEMI. Its ability to quantify hemodynamic overload through noninvasive imaging offers a promising method for identifying individuals at higher risk of adverse outcomes. However, while VExUS appears to be useful in predicting mortality by capturing degrees of systemic congestion, its role in evaluating AKI in STEMI may be limited. This limitation likely stems from the multifactorial pathophysiology of AKI in this context, which involves not only venous congestion but also ischemic renal injury, contrast-induced nephropathy, and various systemic inflammatory and hemodynamic factors.^2,3^ Therefore, relying exclusively on VExUS to predict AKI may overlook important contributors to renal dysfunction in STEMI, reducing its utility for this specific outcome. Our findings differ from those of Viana-Rojas et al.^4^ likely due to differences in study populations. While their analysis included individuals with unstable angina and NSTEMI, our study focused solely on STEMI. Nevertheless, both studies support the use of altered VExUS scores as meaningful risk markers for adverse outcomes in ACS. Although other ultrasound-based tools — such as lung ultrasound and left ventricular outflow tract velocity time integral^7,8^ — may offer superior performance in identifying high-risk individuals when compared with clinical evaluation alone, our findings suggest that integrating VExUS with the Killip classification further enhances predictive accuracy, as evidenced by an improved AUC. This indicates that VExUS may serve as a valuable adjunct in early risk stratification, especially in the setting of right ventricular MI. However, this hypothesis warrants further investigation and validation in larger cohorts, particularly those with a higher prevalence of right ventricular involvement.

This study has several limitations. First, its single-center design and relatively small sample size may have limited the statistical power and the accuracy of the regression model. However, it is important to note that this was a registry of consecutive and unselected individuals with STEMI admitted to a tertiary referral center, which enhances the generalizability within similar clinical settings. To date, this is the first and largest study to evaluate the VExUS score specifically in individuals with STEMI and the first to demonstrate an association between VExUS and in-hospital mortality in this population. Still, future multicenter studies are needed to confirm and expand upon these findings.

## Conclusion

VExUS appears to be a promising tool for identifying high-risk individuals and predicting in-hospital mortality following STEMI. These findings underscore the value of comprehensive hemodynamic assessment in the early prognostic evaluation of this population.

## Data Availability

All datasets supporting the results of this study are available upon request from the corresponding author Guilherme Pinheiro Machado. The dataset is not publicly available because it contains information that could compromise the privacy of research participants.
